# *Fuento*: functional enrichment for bioinformatics

**DOI:** 10.1093/bioinformatics/btx179

**Published:** 2017-03-30

**Authors:** David Weichselbaum, Bojan Zagrovic, Anton A Polyansky

**Affiliations:** Department of Structural and Computational Biology, Max F. Perutz Laboratories & University of Vienna, Campus Vienna Biocenter 5, Vienna, Austria

## Abstract

**Summary:**

The currently available functional enrichment software focuses mostly on gene expression analysis, whereby server- and graphical-user-interface-based tools with specific scope dominate the field. Here we present an efficient, user-friendly, multifunctional command-line-based functional enrichment tool (*fu-en-to*), tailored for the bioinformatics researcher.

**Availability and Implementation:**

Source code and binaries freely available for download at github.com/DavidWeichselbaum/fuento, implemented in C ++ and supported on Linux and OS X.

## 1 Introduction

A growing body of knowledge concerning biomolecular sequences, functions and structures provides a platform for computational studies aimed at linking the properties of sequences with relevant functional categories. Any time these studies result in a metric suitable for classification, a powerful method for validating its biological relevance entails calculating non-random enrichment of gene functions/classes by testing for statistical significance with the aid of e.g. Fisher's Exact Test or hypergeometric and binomial tests (Rice, 2007). These statistical methods provide the probability of encountering a given function at a certain frequency by chance in a subset of genes taken from a larger, annotated background set.

**Fig. 1:**
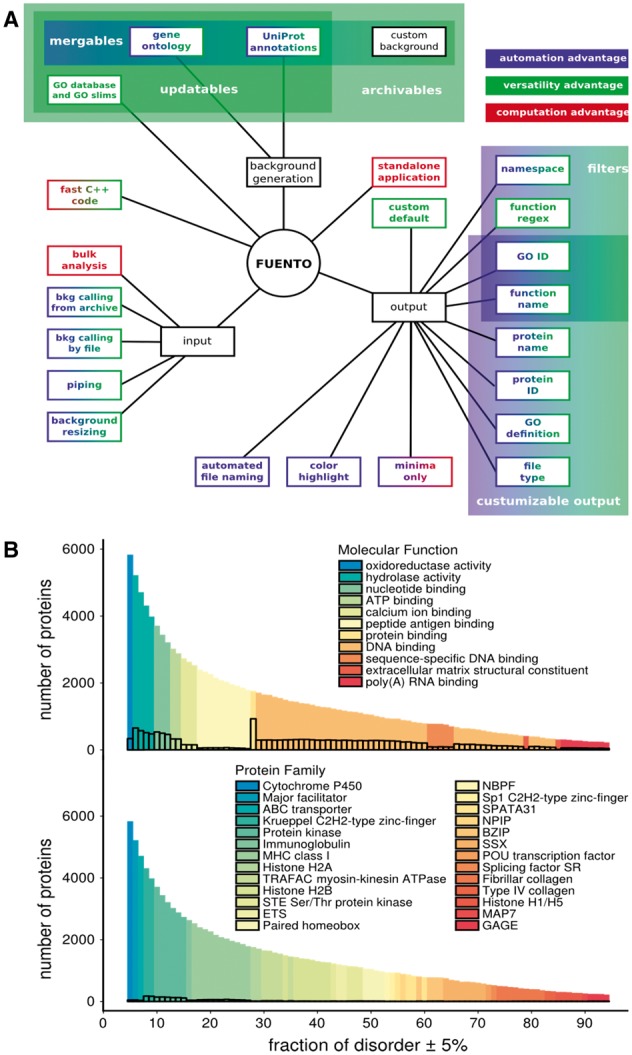
Advantages and exemplary results of *fuento*. (**A**) Select features of *fuento*, categorized as background (bkg) generation, output and input, with colored-coded comparative advantages. The shaded boxes describe options which apply to a range of arguments. (**B**) Structural disorder spectra of molecular functions and families of human proteins. The distributions correspond to the number of proteins in a given set, colored according to the most enriched function. Framed bars represent the number of proteins in each set annotated with the most enriched functions

Previously chiefly a tool for analysis of microarray data, functional enrichment analysis (FEA) has grown to allow probing of different gene/protein lists from various ‘-omics’ experiments. However, the majority of tools available for functional enrichment analysis are web-based and focus on specific contexts (Shi Jing *et al.*, 2015). A few notable web-tools are: PANTHER (Mi *et al.*, 2013), a tool for FEA of protein functions and heritage; DAVID (Huang *et al.*, 2009), which enables clustering on a wide range of functional annotation; Enrichr (Chen *et al.*, 2013), which provides a variety of visualization options; g:Profiler (Reimand *et al.*, 2016), which is tied in with different annotation web-services; and GOrilla (Eden *et al.*, 2009), which produces visual representations of functional graphs. The existing stand-alone tools (e.g. g:Profiler) are mostly GUI-centric and are not suitable for batch analysis, lack advanced settings and tie in poorly or not at all with shell-scripts. Specifically, a number of functional enrichment R-scripts are available in Bioconducor (Gentleman *et al.*, 2004) as well as in dedicated packages (e.g. topGO, gage (Mi *et al.*, 2013), Gostats (Falcon and Gentleman, 2007)) but they are relatively slow, often specialized, have many dependencies and do not optimally tie in with downstream processing. Command-line tools exist, but they are often by-products of web-based services or are library-based and, therefore, lack stand-alone application features. This is less crucial when working with ‘-omics’ data, since analysis is the least time-consuming step and a high involvement can be expected from the user. Bioinformatic analysis, however, prioritizes speed, automation of annotation, flexible filters and arguments, tunability of output, richness of the command-line API and user-friendliness when used as a stand-alone application. To fill this gap, we here present *fuento*, the functional enrichment tool, a stand-alone command-line application for functional enrichment analysis. It is designed both for speedy and automated analysis tied in with shell scripts, as well as for rapid inspection of gene sets with a minimal number of commands. *Fuento’s* comparative advantages when it comes to automation (diverse filters, file handling), versatility (background generation and updating, customizable standard output) and computation (bulk analysis, stand-alone application) are given in Figure 1A.

## 2 Workflow and implementation

A statistical test is used to assign rank scores to over- or under-represented categories between sets of items. In classical enrichment analysis, the compared sets are two lists of genes, a background to be tested against (e.g. a genome) and a subset thereof resulting from some analysis. *Fuento* applies its own, fast C ++ implementations of the one-sided Fisher's Exact Test as well as hypergeometric and binomial tests using two dynamically generated buffers, one for log-factorials used in the calculation of p-values and one for results already generated for the same distribution of categories/items. These speedups are especially powerful in bulk analysis, a capability often neglected by other enrichment tools. The p-values are calculated for each category represented more than once in the background. By default, *fuento* uses a permutation test to find a cutoff for the displayed function by generating 100 random sets from the background with the same size as the test set and calculates the average lowest probability. Since thousands of probabilities are generated, multiple hypothesis correction needs to be applied. In *fuento*, we have implemented the Bonferroni method (Bonferroni *et al.*, 1936) together with two false-discovery rate (FDR) controlling methods, Benjamini-Hochberg FDR correction (Benjamini and Hochberg, 1995) and Benjamini–Hochberg–Yekutieli FDR adjustment (Yekutieli and Benjamini, 1999). The order and type of tests as well as their sorting, corrections and color highlighting can be specified, but sensible defaults facilitate rapid analysis. These defaults are customizable via an argument. The categorical data used in *fuento* can be functional annotations such as those curated by Gene Ontology (The Gene Ontology Consortium, 2000), gene families, motives, localization data or any of the 172 UniProt (The UniProt Consortium, 2012) annotation types. *Fuento* is capable of generating backgrounds with the desired categories from the above-mentioned online sources using files of gene IDs. *Fuento* automatically maps to 99 supported gene ID types. Because this online resources update their annotations in a monthly fashion, *fuento's* backgrounds and databases can be automatically kept up-to-date with a single command. The background format is flexible and can include not only gene IDs annotated with gene-ontology IDs, but any item followed by any annotation in plain text. Such flexibility makes *fuento* a universal functional enrichment tool. The sourcecode is written in C ++ and uses stdlib together with boost (Abrahams and Gurtovoy, 2002) and cURL (curl.haxx.se) libraries.

## 3 Performance

To demonstrate *fuento*’s strong points, we employ the tool to study how protein functions depend on their structural disorder. IUpred (Dosztányi *et al.*, 2005) is used to calculate the probability for a residue in a given sequence to be disordered for each member of a set of 17856 human proteins, generated from UniProt entries with an evidence level higher than ‘uncertain’, containing full coding sequences (Hlevnjak *et al.*, 2012). The fraction of ‘disordered residues’ for each protein is estimated by treating all residues with a disorder probability >0.5 as disordered (Dosztányi *et al.*, 2005). Proteins are grouped in equally spaced sets with disorder fraction ≥5% and ≤95%, incremented by 1%, so that each set is comprised of proteins with the same fraction of disordered residues ±5%. Background files are generated automatically from files of UniProt IDs. The *fuento*'s ‘create gene ontology background’ and ‘create background from UniProt knowledge base’ arguments download correct annotations using the EBI service QuickGO (Binns *et al.*, 2009) and UniProt online resources, respectively. Overall, backgrounds were generated and archived in a matter of minutes and merged into one file for convenience. Analysis was done by running *fuento* in bulk mode over all sets with a filter for the maximally enriched function and the respective function namespaces. On a standard desktop machine, the analysis took under 10 seconds for 100 subsets of human proteins, which is approximately 60 times faster than the most comparable command-line tool (Ontologizer (Bauer, 2008)). Here molecular functions and protein families exhibit preferences for certain regions of disorder, with the most folded proteins corresponding to metabolism followed by membrane transport and translational control, while the most disordered proteins group around RNA-related functions (Fig. 1B).

To summarize, *fuento* was developed for fast, facile and flexible functional enrichment analysis, which we demonstrated on the example of a large-scale exploration of the functional correlates of protein disorder. The tool and its documentation are available at GitHub (github.com/DavidWeichselbaum/fuento).

## Funding

The support by an ERC Starting Independent grant Nr. 279408 to BZ is gratefully acknowledged.

*Conflict of Interest*: none declared.
